# Development and Evaluation of Multifunctional Poly(Lactic-co-glycolic acid) Nanoparticles Embedded in Carboxymethyl β-Glucan Porous Microcapsules as a Novel Drug Delivery System for Gefitinib

**DOI:** 10.3390/pharmaceutics11090469

**Published:** 2019-09-12

**Authors:** Xiaonan Li, Jinglei Wang, Shang Li, Zhaorong Liu, Zhiru Zheng, Yanzhuo Zhang

**Affiliations:** 1 Jiangsu Key Laboratory of New Drug Research and Clinical Pharmacy, Xuzhou Medical University, Xuzhou 221004, China; xiaonanlike@126.com (X.L.); lishang95@126.com (S.L.); 2 Department of Pharmaceutics, School of Pharmacy, Xuzhou Medical University, Xuzhou 221004, China; jinglei94@126.com (J.W.); liuzhaorong123@126.com (Z.L.); xuyynt@126.com (Z.Z.)

**Keywords:** nanocomposites, controlled delivery, dual-particulate, cellular uptake, carboxymethyl β-glucan, chemotherapeutic drugs

## Abstract

In this study, a new kind of folic acid (FA)-conjugated and chitosan (CS)-coated poloxamer 407 (P407)/poly(lactic-*co*-glycolic acid) (PLGA) nanoparticles (NPs), FCPP NPs, were prepared, and further micro-encapsulated by carboxymethyl β-glucan microcapsules (MCs) to produce a multifunctional system of NPs embedded in MCs (NEMs) for potential lung tumor-targeted delivery of gefitinib. The prepared gefitinib-loaded FCPP (GFB/FCPP) NPs showed a hydrodynamic diameter of 255.4 ± 14.5 nm and existed in an amorphous state. After encapsulation in carboxymethyl β-glucan MCs, the GFB/FCPP-based NEMs (GFB/FCPP-NEMs) also exhibited a spherical morphology with a median diameter (d_50_) of around 2.2 μm. After hydration, GFB/FCPP- NEMs can quickly dissociate into its primary particles, GFB/FCPP NPs. Our in vitro drug release study revealed that these GFB/FCPP-NEMs exhibited a pH-responsive prolonged release property. In addition, the cellular uptake study demonstrated that FCPP-NEMs serve as an efficient carrier to enhance the delivery of the entrapped drug into the target lung tumor cells. Moreover, the GFB/FCPP-NEMs induced a superior cytotoxic effect compared with free gefitinib. The inhibitory concentration to achieve 50% cell death (IC_50_) of GFB/FCPP-NEMs in A549 cells was 3.82-fold lower than that of free gefitinib. According to these results, FCPP-NEMs hold a great potential as a multifunctional and high-performance biomaterial for lung tumor targeting delivery, pH-responsive sustained release, facilitated cellular uptake, and enhanced antitumor effect of antitumor drugs, like gefitinib.

## 1. Introduction

According to the 2018 report of the World Health Organization (WHO), lung cancer, particularly non-small cell lung cancer (NSCLC), is the leading cause of cancer-related human death worldwide. Until now, depending on the cancer cell type and stage, lung cancer has been treated primarily by chemotherapy, radiotherapy, surgery or a combination of them [1,2]. However, due to the high toxicity of most anticancer drugs, undesirable tissue distribution, inadequate penetration, and rapid clearance from blood circulation, chemotherapy often has in low efficacy, severe systemic toxicity and adverse effects [3,4].

Gefitinib is a tyrosine kinase inhibitor that targets the epidermal growth factor receptor (EGFR), which is overexpressed in lung, colon, breast, brain, and ovarian tumors. Due to the dramatic clinical response to gefitinib in NSCLC, gefitinib was approved by the United States (US) Food and Drug Administration (FDA) in 2015 as the first-line treatment for patients with NSCLC [3]. However, a high dose (250 mg/day) of gefitinib is required for its commercial tablets due to its low aqueous solubility, high P-glycoprotein efflux, as well as first-pass metabolism [5,6]. Consequently, oral gefitinib treatment leads to dose-related adverse effects, such as gastrointestinal disorders, nutritional as well as metabolism imbalances, and even, interstitial lung disease, which is fatal in some patients [7]. All the above-mentioned issues indicate that there is a need to design a novel approach or more effective and safer formulation, for the delivery of gefitinib, preferably as a pulmonary inhalant. Pulmonary inhalant would target delivery of therapeutics to the lungs with effective drug concentrations, and afford rapid onset of therapeutic action as well as minimal systemic side effects [8].

Over the last decade, the biodegradable and body-friendly polymeric micro/nanoparticles (NPs) have played an important role in cancer treatment. Among the various polymeric materials, poly(lactic-*co*-glycolic acid) (PLGA), a pharmaceutical excipient in the US Pharmacopeia and National Formulary (USP/NF) monograph, has become very popular in drug delivery systems due to its modified particle sizes, stable solid structure, facile surface functionalization, and excellent biocompatibility and biodegradability [9,10]. As a result of their stable micro/nanoscale solid structure, PLGA NPs exhibit an excellent ability to control release of their incorporated drug, which may be of value to prolong the systemic circulation time. More importantly, surface modification of PLGA NPs with functional polymers can confer them additional favorable characteristics. It is well established that chitosan (CS)-coated PLGA NPs have enhanced muco-adhesiveness and enabled sustained delivery of peptides to lungs [11]. Recently, Vasudev et al. prepared trimethyl CS chloride- modified PLGA NPs and demonstrated that these NPs combine the controlled release property of PLGA NPs with the muco-adhesive property of trimethyl CS chloride for oral insulin delivery [12]. In addition, Dhas et al. used FA-conjugated CS-coated PLGA NPs for delivery of bicalutamide in prostate cancer [13]. Unlike the above-mentioned surface modification, some reports have described the incorporation of a functional polymer into PLGA NPs. For example, Zhu et al. used a co-matrix of PLGA and D-tocopherol polyethylene glycol (1000) succinate (TPGS) to control the release rate, enhance encapsulation efficiency, and the antitumor effect of docetaxel [14]. Together, these findings suggest that the combination of PLGA and functional polymers may be a suitable strategy to achieve efficient delivery of antitumor drugs.

Therefore, the design and fabrication of a novel FA-conjugated and CS-coated poloxamer 407 (P407)/PLGA NPs (FCCP NPs) co-matrix nanocarrier for gefitinib delivery is highly desirable. This hybridized nanocarrier, FCPP NPs, would combine the unique benefits of FA, CS, PLGA NPs, and P407 into a single carrier system. On the one hand, the FA-conjugated CS affords the FA receptor (overexpressed on the cell surface of many human epithelial tumor cells) targeted feature, muco- adhesive property and pH-triggered release control capacity [15,16]. On the other hand, P407, as a co-matrix agent, can enhance drug permeability by reducing the P-glycoprotein efflux of tumor cell membranes, and thus suppresses the multidrug resistance of the tumor cells [17].

However, despite the multiple attractive advantages of FCPP NPs, nanoscale particles are not suitable for efficient pulmonary inhalation delivery [8,18]. To address this issue, it is necessary to encapsulate these FCPP NPs into soluble and biodegradable porous microcapsules (MCs, specifically 1–5 μm) that can effectively deposit in the lungs, and then dissociate to their primary particles (FCPP NPs) for tumor-targeted delivery. Among the various available MC materials, carboxymethyl β- glucan (a water-soluble β-glucan derivative) may be an excellent candidate due to its high biocompatibility, biodegradability, and bioadhesion [19]. More importantly, carboxymethyl β-glucan is a polysaccharide, which has well-known immunomodulatory properties, including direct activation of NK cells and cytokine release by specialized phagocytes that stimulate phagocytosis [20]. Quite recently, a new approach using resveratrol and carboxymethyl β-glucan has been developed to treat respiratory diseases [21]. Accordingly, these findings make it very tempting to undertake the fabrication of a multifunctional biocompatible and stable NEM system for lung- targeted antitumor drug delivery via the encapsulation of these FCPP NPs into carboxymethyl β-glucan MCs, which, to the best of our knowledge, has not been reported to date.

Therefore, in this study, we sought to develop such a novel type of FCPP-NEM carrier system to achieve effective lung tumor-targeted delivery, pH-responsive sustained release, facilitated cellular uptake, and enhanced antitumor effects of the antitumor drugs, like gefitinib. To achieve this goal, we systematically evaluated the physicochemical properties of the newly developed GFB/FCPP- NEMs, including morphology, particle size distribution (PSD), crystalline state, drug loading amount, Fourier transform infrared (FT-IR) spectrum, drug release profiles, and release mechanism. In addition, besides studying the cellular uptake of FCPP NPs (the primary particles of FCPP-NEMs), the cytotoxicity of GFB/FCPP-NEMs was evaluated in the lung cancer cell lines A549 and PC-9. Additionally, an accelerated stability study was conducted to determine the physicochemical stability of GFB/FCPP-NEMs.

## 2. Materials and Methods

### 2.1. Materials

P407 was obtained from BASF SE (Ludwigshafen, Germany). Carboxymethyl β-glucan was purchased from Yuancheng Chemical Co., Ltd. (Suzhou, China). PLGA (Mw = 24,000–38,000, lactide/glicolide = 50/50) was provided by Daigang Biomaterials (Jinan, China). USP grade gefitinib (purity ≥99%) was obtained from Yadong Pharma Ltd. (Nanjing, China). FA, polyvinyl alcohol (PVA), and CS (Mw = 8000 Da, degree of deacetylation ≥75%) were purchased from Aladdin Reagent Co., Ltd. (Shanghai, China). High-performance liquid chromatography (HPLC) grade acetonitrile and methanol were purchased from Sinopharm Chemical Reagent Co., Ltd. (Shanghai, China). The other chemicals and reagents were of analytical grade.

### 2.2. Cell Culture

The human NSCLC cell lines A549 and PC-9 were obtained from the Chinese Academy of Sciences (Shanghai, China). A549 and PC-9 cells were cultured in high glucose Dulbecco’s modified Eagle’s medium (DMEM) and Roswell Park Memorial Institute (RPMI)-1640 medium, respectively. Both culture media were supplemented with antibiotic (100 U/mL penicillin and 100 µg/mL streptomycin) and 10% fetal bovine serum. The cells were cultured under humidified conditions at 37 °C and 5% CO_2_.

### 2.3. Fabrication of GFB/FCPP NPs

**Figure 1:**
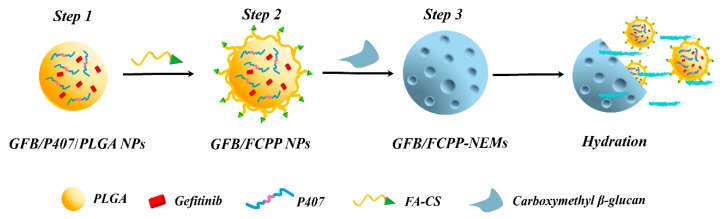
Schematic diagram of the preparation of gefitinib-loaded and folic acid (FA)-conjugated chitosan (CS)-coated poloxamer 407 (P407)/poly (lactic-co-glycolic acid) (PLGA) nanoparticles (NPs), namely GFB/FCPP NPs and GFB/FCPP NPs embedded in microcapsules (MCs), namely GFB/FCPP- NEMs.

The monodispersed GFB/FCPP NPs were prepared by a two-step process: First, the gefitinib- loaded P407/PLGA (GFB/P407/PLGA) NPs were fabricated by a double emulsion–solvent evaporation method (Step 1 in Figure 1) [22]. Briefly, 20 mg of P407, 10 mg of gefitinib, and 80 mg of PLGA were dissolved in 7 mL of dichloromethane (DCM) to obtain the organic phase. Subsequently, 2 mL of distilled water, used as the aqueous phase, was poured into the above organic phase, and mixed by stirring at 12,000 rpm for 6 min to obtain a coarse emulsion. Then, the coarse emulsion was sonicated with an ultrasonic probe for 6 min (pulsing mode 3 s on and 2 s off, 300 Walt) in an ice water bath. Next, the resulting emulsion was poured into 30 mL of a 1% PVA aqueous solution and the mixture was further sonicated for 6 min in an ice water bath. After sonication, the resulting double emulsion was stirred overnight using a propeller-type agitator at 200 rpm to remove the DCM. Afterwards, the suspension was centrifugated at 9000 rpm for 15 min, washed with distilled water and the GFB/P407/PLGA NPs were collected.

In the subsequent step, the obtained GFB/P407/PLGA NPs were coated with FA-conjugated CS and then lyophilized to form the GFB/FCPP NPs (Step 2 in Figure 1). Briefly, FA conjugated CS was prepared based on a carbodiimide reaction as previously reported (Supplementary Materials) [23]. Then, 0.5 g of GFB/P407/PLGA NPs was resuspended in 10 mL of the FA-conjugated CS solution (1%, in 1% acetic acid solution). Next, the resulting mixture was vortexed for 5 min and stirred for 3 h. After centrifugation at 9000 rpm for 20 min, the collected GFB/FCPP NPs were washed with distilled water, and lyophilized at −55 °C for 10 h using a laboratory-scale freeze-drying device (Evela Instruments, Tokyo, Japan) at a pressure below 10 Pa. Ultimately, the lyophilized powder was passed through a 80-mesh sieve, and stored in a screw cap sealed glass bottle.

### 2.4. Fabrication of GFB/FCPP-NEMs

The encapsulation of GFB/FCPP NPs in carboxymethyl β-glucan MCs was carried out by the well-known spray drying method (Step 3 in Figure 1). In a typical preparation, 1 g of carboxymethyl β-glucan was dissolved in 100 mL of purified water to form a 1% carboxymethyl β-glucan aqueous solution. Then, 0.4 g GFB/FCPP NPs was dispersed in the above solution under magnetic stirring until a uniform dispersion was formed. Eventually, the dispersion was pumped into an ADL-310 mini bench-top spray dryer (Nanjing, China) to obtain the GFB/FCPP-NEMs. The inlet and outlet temperatures of the drying chamber were set to 170 and 60 °C, respectively. The atomization pressure was set to 170 kPa. The drying air flow rate and feed rate were 0.6 m^3^/min and 5.0 mL/min, respectively. The GFB/FCPP NPs dispersion was continuously stirred during the drying process. Finally, the resulting dry powder was stored in a screw cap sealed glass bottle.

### 2.5. Characterization of the GFB/FCPP NPs and GFB/FCPP-NEMs

#### 2.5.1. Morphological Analysis

Particle shape and surface morphology of the powdered GFB/P407/PLGA NPs, GFB/FCPP NPs, and GFB/FCPP-NEMs were examined by scanning electron microscopy (SEM) using a Teneo VS™ scanning electron microscope (FEI Co., Hillsboro, OR, USA). Before SEM analysis, a small section of each sample was sputtered with a thin gold/palladium layer for conductivity.

#### 2.5.2. Particle Size Distribution and Zeta Potential Measurements

For the prepared GFB/P407/PLGA NPs and GFB/FCPP NPs, the mean hydrodynamic diameter, polydispersity index, and surface charge were measured using a Nicomp^TM^ 380 ZLS zeta potential/size analyzer (Particle Sizing Systems, Santa Barbara, CA, USA). Prior to measurements, each sample was appropriately diluted and highly dispersed in distilled water. For the prepared GFB/FCPP-NEMs powder, laser diffraction was used to determine the particle size distribution using a WJL-612 laser diffraction particle size analyzer (Rise Instruments Co., Ltd., Nanjing, China). The particle size was expressed as the median diameter (d_50_). The span values = [d_90_−d_10_]/d_50_, where d_90_, d_50_ and d_10_ indicate the equivalent volume diameters corresponding to the 90%, 50%, and 10% points of the cumulative distribution curve, respectively. All the measurements were performed in triplicate for each sample.

#### 2.5.3. Solid-State Test

The thermal behavior of crude gefitinib, GFB/P407/PLGA NPs, physical mixture of gefitinib and P407/PLGA NPs, GFB/FCPP NPs, carboxymethyl β-glucan, and GFB/FCPP-NEMs was analyzed by differential scanning calorimetry (DSC) using an NY-101B, differential scanning calorimeter (Netzyu Electronics Technology Co., Ltd., Shanghai, China). Powder samples (approximately 10–16 mg) were accurately weighed, placed in ceramic crucibles with a lid under a constant purge of nitrogen at 40 mL/min. Each sample was scanned from 40 to 300 °C (at a ramp rate of 10 °C/min).

#### 2.5.4. FT-IR Spectroscopy Analysis

FT-IR spectra of the FA conjugated CS, crude gefitinib, P407/PLGA NPs, physical mixture of gefitinib and P407/PLGA NPs, GFB/FCPP NPs, and GFB/FCPP-NEMs were recorded using a Bruker IFS 55 FT-IR spectrometer (Bruker Optics GmbH, Ettlingen, Germany). Before measurement, each sample was ground and pressed into potassium bromide discs.

#### 2.5.5. Drug Loading Amount Measurements

Gefitinib content was determined by HPLC analysis using the Agilent 1200 series HPLC system (Agilent Technologies, Santa Clara, CA, USA), which consisted of a tunable UV detector, an autosampler, and a column heater (set at 35 °C). The stationary phase was a reverse phase Agilent Zorbax Eclipse Plus C18 column, while the mobile phase was a mixture of acetonitrile and 10 mM ammonium dihydrogen phosphate buffer (51:49, *v*/*v*). To determine the drug loading amount and entrapment efficiency (EE) of each gefitinib formulation, a predetermined amount of GFB/FCPP NPs or GFB/FCPP-NEMs powder was added in 4 mL of a mixture solution (DCM: acetonitrile, 50/50), and continuously vortexed for 10 min to ensure complete release of the encapsulated gefitinib. Subsequently, the solvent was evaporated under nitrogen. Then, 4 mL of acetonitrile was added to the extract and vortex mixed for 2 min followed by agitation overnight to dissolve the gefitinib. After centrifugation at 9000 rpm for 5 min, the supernatant was collected for HPLC analysis. The EE was defined as: the amount of gefitinib in the samples ×100%/total amount of gefitinib added during the fabrication of the samples. The drug loading amount was expressed as: total amount of gefitinib in the samples ×100%/the amount of the samples.

#### 2.5.6. In Vitro Drug Release Test

A dialysis bag diffusion method was used to examine the drug release behaviors of GFB/FCPP NPs, and GFB/FCPP-NEMs under two different pH values. Briefly, raw gefitinib, GFB/FCPP NPs, or GFB/FCPP-NEMs with equal amount of gefitinib (4 mg) were individually suspended in 2 mL of phosphate-buffered solution (PBS, pH 7.4) containing 1% Tween 80 and sealed in a pre-swelled cellulose acetate dialysis bag. Then, the dialysis bag was immersed in another 100 mL of PBS and shaken horizontally at 100 rpm and 37 °C for 48 h. Tween 80 was used to maintain the sink conditions. At the scheduled time intervals, samples of approximately 4.0 mL were collected from the release medium and replaced with fresh PBS. Each sample was filtered (through a 0.45-μm membrane), transferred into a vial and the released amount of drug was determined by HPLC analysis. Additionally, to evaluate the pH-responsive controlled release property of GFB/FCPP NPs and GFB/FCPP-NEMs, the above-mentioned analysis was performed with pH 5.0 PBS as another release medium. Different kinetic models of zero-order, first-order, Higuchi, and Korsmeyer–Peppas were used to investigate potential release mechanisms of GFB/FCPP-NEMs [24].

### 2.6. Cellular Uptake Study

The primary particles of FCPP-NEMs, namely FCPP NPs, taken up by A549 cells were determined by fluorescence microscopy analysis. Coumarin-6 was used to visualize FCPP NPs. The preparation method for coumarin-6-loaded FCPP NPs was the same as that used for GFB/FCPP NPs except that gefitinib was replaced with coumarin-6. For cellular uptake experiments, A549 cells were seeded on cover slips at a density of 1 × 10^5^ cells/well in 12-well plates and incubated for 24 h. After removal of the culture medium, A549 cells were incubated with 1 mL DMEM containing coumarin- 6-loaded FCPP or coumarin-6-loaded P407/PLGA samples. Following incubation for 4 h, the A549 cells on the cover slips were washed with PBS, fixed with 4% paraformaldehyde for 10 min at room temperature, and then washed again with PBS. After staining with Hoechst 33,342, the A549 cells were washed with PBS. Then, the cover slip containing A549 cells was covered with 50% glycerol and visualized by fluorescence microscopy using an Olympus IX73 fluorescence microscope (Olympus Corp., Tokyo, Japan).

### 2.7. Cytotoxicity Evaluation of GFB/FCPP-NEMs

The in vitro anticancer activity of GFB/FCPP-NEMs was evaluated in two lung cancer cell lines (A549 and PC-9 cells) by the colorimetric MTT (3-(4,5-dimethylthiazol-2-yl)-2,5-diphenyltetrazolium bromide) assay. Briefly, A549 and PC-9 cells were seeded in 96-well plates at the density of 1 × 10^4^ cells/well in 100 μL of normal culture medium, and cultured overnight. Afterwards, the culture medium was replaced with 100 μL of DMEM or RPMI-1640 containing serial concentrations of gefitinib or GFB/FCPP-NEMs. The graded concentration of gefitinib ranged from 0.2 to 20 μg/mL. After incubation at 37 °C for 48 h, the treatment medium was aspirated from the wells, and replaced with 100 μL of fresh culture medium containing the MTT solution (20 μL, 5 mg/mL). After incubation for 4 h, the MTT solution was aspirated, and the formed formazan crystals were solubilized with 100 μL of dimethyl sulfoxide. Next, the absorbance of the resulting solution was detected at 490 nm using a microplate reader (BioTek, Winooski, VT, USA). Cells treated with normal medium served as the control cells. The cell viability was calculated as follows: optical density (OD) of test cells ×100%/OD of control cells. Additionally, empty FCPP-NEMs were also tested by this procedure.

### 2.8. Physicochemical Stability

To evaluate the physicochemical stability of GFB/FCPP-NEMs, an accelerated stability analysis was performed under the accelerated conditions currently recommended by the International Conference on Harmonization guideline. The GFB/FCPP-NEMs were stored in a screw cap sealed glass bottles in a humidity chamber (75% RH) at constant temperature (40 °C). At the specified time points (1, 2, and 3 months), their particle size, physical state, drug content and related substances of gefitinib were determined.

### 2.9. Statistical Analysis

All results were expressed as the mean ± standard deviation (SD) of three independent experiments. All statistical analyses were performed using the SPSS, predictive analysis software (PASW), Statistic Software (SPSS/IBM Corp., Armonk, NY, USA). Comparison between groups was performed by Student’s *t*-tests. *P*-values <0.05 were considered statistically significant [15].

## 3. Results and Discussion

### 3.1. Preparation and Characterization of GFB/FCPP NPs and GFB/FCPP-NEMs

**Figure 2:**
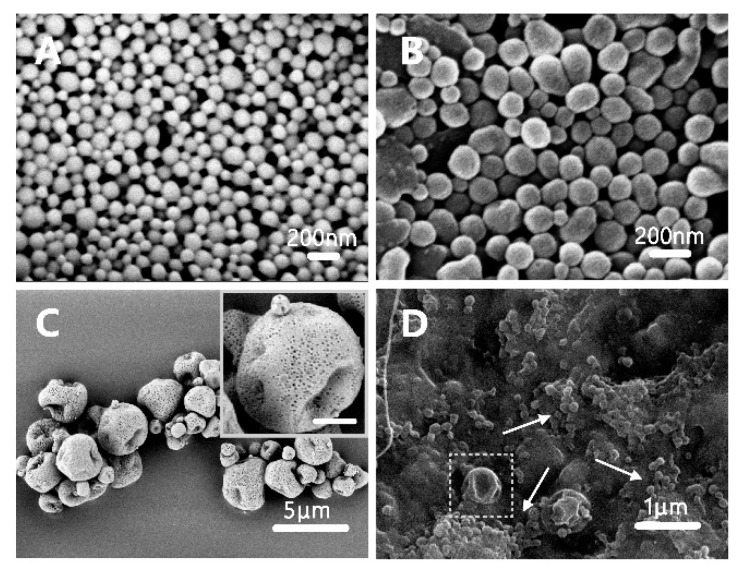
Scanning electron microscopy (SEM) photomicrographs of (**A**) GFB/P407/PLGA NPs, (**B**) GFB/FCPP NPs, (**C**) GFB/FCPP-NEMs (inset, high magnification image, scale bar =1 μm), and (**D**) GFB/FCPP-NEMs after exposure to PBS (pH 7.4). The arrow is pointing to the GFB/FCPP NPs, while the square frame contains a GFB/FCPP-NEM.

The processes for the fabrication of monodispersed GFB/FCPP NPs and GFB/FCPP-NEMs are depicted in Figure 1. In the first process, the GFB/FCPP NPs, which serve as nanoscale drug reservoirs, were prepared by an emulsion–diffusion solvent evaporation process followed by coating with FA-conjugated CS. As shown in Figure S1, the gefitinib crystals consisted of irregularly shaped and dozens of micrometer-sized particles. Unlike gefitinib crystals, the prepared GFB/P407/PLGA samples consisted of a large number of regular spherical NPs with smooth surface without any noticeable cracks (Figure 2A). Moreover, hardly any gefitinib crystals was visible in the GFB/P407/PLGA sample, suggesting that most gefitinib molecules were encapsulated in the P407/PLGA NPs, which was critical for attaining sustained deliver, enhanced cellular uptake, and thereby improving the cytotoxicity of the encapsulated gefitinib molecules [9,24,25]. As shown in Figure 2B, after the FA-conjugated CS coating (Step 2 in Figure 1), the degree of sphericity of the GFB/FCPP NPs was reduced and their surface was relatively coarse compared with that of the GFB/P407/PLGA NPs. The hydrodynamic diameter, surface charge and drug loading amount of the prepared NPs are listed in Table 1. Based on the dynamic light scattering results, the GFB/FCPP NPs had a mean size of 255.4 ± 14.5 nm with a low polydispersity index, suggesting that the GFB/FCPP NPs were monodispersed. The drug loading amount and EE for GFB/FCPP NPs were 7.68 ± 0.36% and 95.57 ± 1.33%, respectively. GFB/FCPP NPs exhibited nanoscale sizes and a narrow size distribution, but nanoscale particles are not suitable for efficient pulmonary delivery. In order to achieve an effective deposition of the gefitinib formulation in deep lung regions (with huge inner surface area), the diameter of the powder particles is required to range from 1 to 5 μm [18]. Thus, the biodegradable and biocompatible polymer excipient, carboxymethyl β-glucan, was used as the MC material and the commonly used spray-drying technique was used for the encapsulation of GFB/FCPP NPs in carboxymethyl β-glucan MCs (Step 3 in Figure 1). Remarkably, as shown in Figure 2C, the formed GFB/FCPP-NEMs were nearly spherical capsules with a highly porous and sunken surface (their pore size was around 20 nm, inset of Figure 2C). The median diameter (d_50_), d_10_, d_90_ and span of GFB/FCPP-NEMs were about 2.2 ± 0.1 μm, 1.2 ± 0.1 μm, 4.6 ± 0.3 μm and 1.6 ± 0.2 μm, respectively. Due to their highly porous surface, GFB/FCPP-NEMs can quickly dissociate into its primary particles (GFB/FCPP NPs) for FA-mediated tumor-targeted drug delivery after hydration, as shown in Figure 2D.

### 3.2. Solid-State Characterization

**Figure 3:**
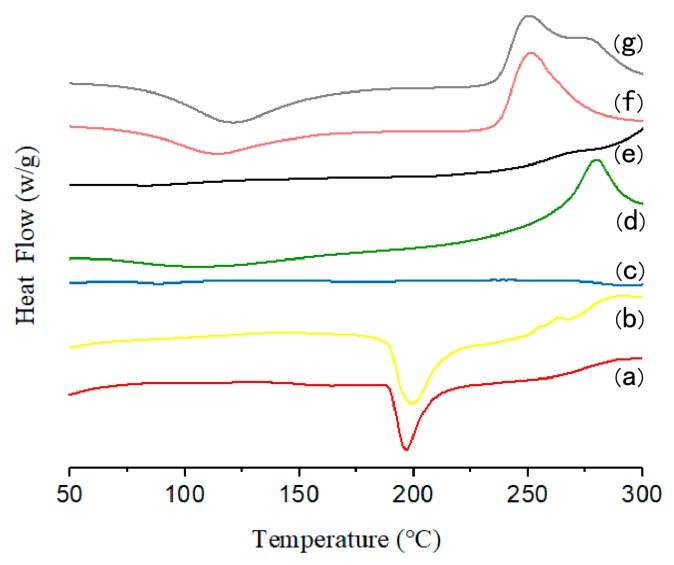
Differential scanning calorimetry (DSC) thermograms of (**a**) gefitinib crystals, (**b**) physical mixture of gefitinib and P407/PLGA NPs, (**c**) GFB/P407/PLGA NPs, (**d**) FA-conjugated CS, (**e**) GFB/FCPP NPs, (**f**) carboxymethyl β-glucan, and (**g**) GFB/FCPP-NEMs.

Thermal analysis has been widely used for determination of the physical state of drugs in the NPs or MCs. Thus, DSC analysis of pure gefitinib, GFB/P407/PLGA, the physical mixture of gefitinib and P407/PLGA NPs, GFB/FCPP NPs, and GFB/FCPP-NEMs was conducted and the obtained DSC thermograms are presented in Figure 3. As shown in Figure 3a, pure gefitinib crystals show an endothermic peak at around 195 °C (with an enthalpy of 390 J/g), which corresponds to its melting temperature. The DSC curve of the physical mixture (gefitinib and P407/PLGA NPs) shown in Figure 3b also exhibited the sharp peak associated with gefitinib, indicating that gefitinib was still present in a crystalline state. However, after loading gefitinib into P407/PLGA NPs, the endothermic peak of gefitinib is not observed in the DSC thermograms of GFB/P407/PLGA NPs (Figure 3c). The absence of the endothermic peak corresponding to crystalline gefitinib in the thermograms indicated that the gefitinib entrapped in the P407/PLGA NPs had mostly lost its crystallinity [26]. Similarly, the endothermic peak of crystalline gefitinib also disappeared for both the GFB/FCPP NPs and GFB/FCPP-NEMs samples (Figure 3e,g). According to the DSC measurements, the encapsulated gefitinib was mostly in an amorphous state in the GFB/FCPP-NEMs delivery system.

### 3.3. FT-IR Characterization

The FT-IR spectra of CS, FA, and FA-conjugated CS samples are displayed in Figure S2. For the FA sample, the typical band at 1694 cm^−1^ is attributed to the C=O stretching vibration, while the band at 1606 cm^−1^ is the characteristic peak of the amino group in the pteridine ring. In addition, the peak at 1484 cm^−1^ corresponds to the vibration of the benzene ring [23]. For the CS sample, the characteristic absorption peaks around 1619 and 1092 cm^−1^ belong to the N−H and C−O stretching vibrations, respectively. After conjugation of FA with CS, the absorption peak at 1514 cm^−1^ was weakened as a result of the reaction of the amino group of CS with the carboxylate group of FA. In addition, the appearance of a new peak at 1606 cm^−1^, which corresponds to the amino group in the pteridine ring of FA, also confirmed that FA was conjugated with CS.

**Figure 4:**
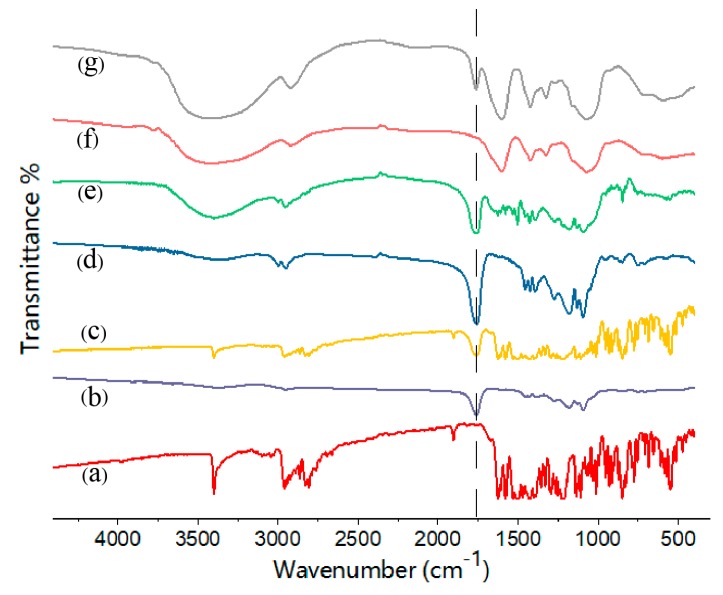
Fourier transform infrared (FT-IR) spectra of (**a**) gefitinib crystals, (**b**) P407/PLGA NPs, (**c**) physical mixture of gefitinib and P407/PLGA NPs, (**d**) GFB/P407/PLGA NPs, (**e**) GFB/FCPP NPs, (**f**) carboxymethyl β-glucan, and (**g**) GFB/FCPP-NEMs.

The FT-IR spectra of pure gefitinib, GFB/P407/PLGA, the physical mixture of gefitinib and P407/PLGA NPs, GFB/FCPP NPs, and GFB/FCPP-NEMs are presented in Figure 4. Pure gefitinib displays several absorption peaks. The FT-IR spectrum of gefitinib shown in Figure 4a reveals a characteristic absorption peak at 1625 cm^−1^ that was attributed to C = N stretching vibrations, while the peaks at 1110 cm^−1^ and 1028 cm^−1^ corresponded to the C−O and C−F stretching vibrations, respectively [27]. As anticipated, these gefitinib absorption peaks are also present in the spectrum of the physical mixture of gefitinib and P407/PLGA NPs (Figure 4c). Compared with the physical mixture, most of the gefitinib characteristic absorption bands were weakened in the FT-IR spectrum of the GFB/P407/PLGA sample (Figure 4d), suggesting that gefitinib was entrapped in the P407/PLGA NPs. After FA-conjugated CS coating, the absorption peaks at 1694, 1606, and 1484 cm^−1^ corresponding to the FA-conjugated CS can be observed in the FTIR spectrum of the GFB/FCPP NPs (Figure 4e). Moreover, the absorption peaks of GFB/FCPP-NEMs (Figure 4g) were similar to those of the blank carboxymethyl β-glucan MCs, except for the intense peak at 1752 cm^−1^ corresponding to the C=O bond of the ester group from PLGA. Combine with the SEM analysis, these results clearly suggest that GFB/FCPP NPs and GFB/FCPP-NEMs were successfully prepared.

### 3.4. Drug Release Profiles

**Figure 5:**
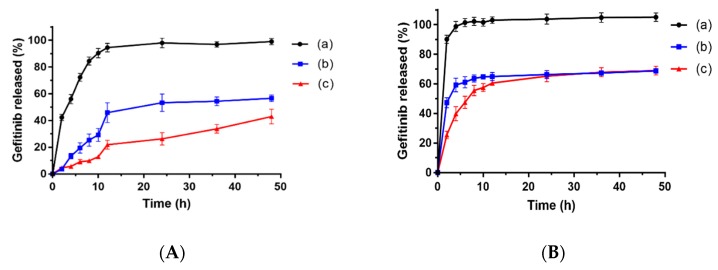
Drug release of different gefitinib formulations at (**A**) pH 7.4 and (**B**) pH 5.0. (**a**) gefitinib suspension, (**b**) GFB/FCPP NPs, and (**c**) GFB/FCPP-NEMs. Each data point represents the mean ± SD (*n* = 3).

In vitro drug release tests were conducted at two different pH values (pH 7.4 and pH 5.0) to simulate the systemic circulation conditions and weakly acidic environments inside the tumor sites, respectively. The results revealed that the release rate of gefitinib from the gefitinib suspension was fast in both media. Even at pH 7.4 (mimic physiological conditions), approximately 60% of gefitinib was released from the gefitinib suspension within 4 h (Figure 5A). Unlike the rapid release pattern of the gefitinib suspension, the release profiles of gefitinib from GFB/FCPP NPs exhibited a slow and prolonged drug release behavior. Only about 13% of gefitinib was released in the initial 4 h, and the cumulative release rate of gefitinib from GFB/FCPP NPs was no more than 60% after 48 h. Compared with gefitinib suspension, the sustained-release behavior of GFB/FCPP NPs can be attributed to the encapsulation of gefitinib into FCPP NPs, which can lessen the damage of gefitinib to normal cells in the human body [28,29]. In addition, it is worth noting that the release of gefitinib from GFB/FCPP- NEMs was slower than that of GFB/FCPP NPs. As shown in Figure S3, after hydration of carboxymethyl β-glucan MCs, a hydrophilic gel of carboxymethyl β-glucan was formed and surrounded the GFB/FCPP NPs, which contributed to the decreased release rate of gefitinib from GFB/FCPP-NEMs. As expected, the release rate of gefitinib from the GFB/FCPP NPs or GFB/FCPP- NEMs at pH 5.0 was significantly fast compared with that at pH 7.4, as shown in Figure 5B. For the GFB/FCPP NPs, the amount of released gefitinib from at the sampling time of 2 h reached approximately 45% and was almost 60% within 4 h at pH 5.0. In addition, nearly 70% of gefitinib was released after 48 h. The enhanced drug release rate of GFB/FCPP NPs can be attributed to the pH-dependent FA-conjugated CS coating. As is well known, CS can be dissolved under acidic conditions. Since tumor microenvironment is weakly acidic, with pH values down to 5.0, the enhanced drug release efficiency of GFB/FCPP NPs at acidic pH would be beneficial in improving the cytotoxic and antitumor effects of the entrapped drug [30,31]. More importantly, GFB/FCPP NPs can release many more drug molecules at tumor sites than at normal tissues and thereby avoid side effects due to universal cytotoxicity. The above results indicated that the prepared FCPP-NEMs are likely an ideal drug delivery system for pH-dependent sustained drug release. Additionally, the probable release mechanism was investigated using different kinetic models of zero-order, first- order, Higuchi, and Korsmeyer–Peppas to fit the drug release curves of GFB/FCPP NPs and GFB/FCPP-NEMs, and their model parameters are summarized in Tables S1 and S2. The results revealed that the gefitinib release from both GFB/FCPP NPs and GFB/FCPP-NEMs was best fitted with the first-order model (with the highest value of regression coefficients, *r*^2^ ≥ 0.96) suggesting diffusion-controlled drug release kinetics. In addition, based on the Korsmeyer–Peppas model, the release exponent (*n*) for the tested GFB/FCPP NPs (at pH 5.0 and 7.4) or GFB/FCPP-NEMs (at pH 5.0) below 0.45, suggesting that the release of gefitinib from the test samples mainly follows the Fickian diffusion mechanism [32,33]. In contrast, the release exponent (*n*) for the tested GFB/FCPP-NEMs at pH 7.4 up to 0.69, indicated that gefitinib release from GFB/FCPP-NEMs was based on a combined diffusion/erosion process [34].

### 3.5. Cell Uptake and Intracellular Localization

**Figure 6:**
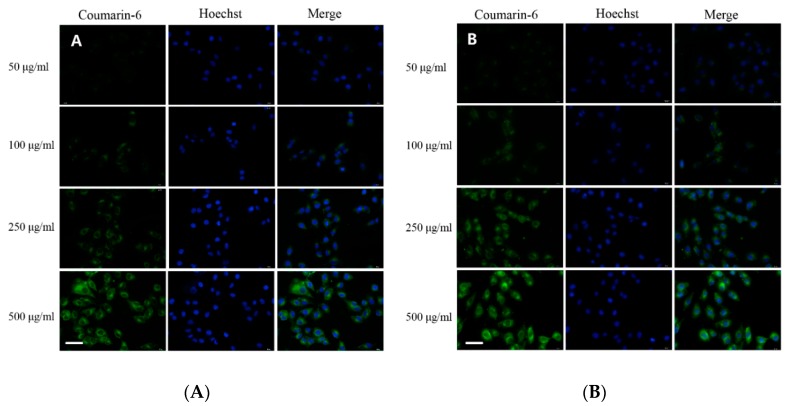
Fluorescence images of A549 cells after incubation with coumarin-6-loaded P407/PLGA NPs (**A**) and coumarin-6-loaded FCPP NPs (**B**). Scale bar = 20 μm.

To confirm the cell uptake of the primary particles of FCPP-NEMs, namely FCPP NPs, a fluorescence imaging analysis was carried out. The fluorescence microscopy images of A549 cells after 4 h incubation with coumarin-6-loaded FCPP NPs are shown in Figure 6. For comparison purposes, the cellular uptake behavior of coumarin-6-loaded P407/PLGA NPs was also studied. As shown in Figure 6A,B, the nucleus of the cells was stained blue with the Hoechst 33,342 dye. Also, obvious green fluorescence can be observed in the cytoplasmic region of cells with both coumarin- 6- loaded NPs tested, indicating that both coumarin-6-loaded FCPP NPs and coumarin- 6- loaded P407/PLGA NPs can be internalized by the A549 cells. Moreover, a significant enhancement in the green fluorescence intensity in the cytoplasmic region was observed with the increase of the dose of each carrier, suggesting that the uptake of both carriers was dose-dependent. As anticipated, compared with coumarin-6-loaded P407/PLGA NPs, cells exposed to coumarin- 6- loaded FCPP NPs had a slightly stronger green fluorescence at concentrations from 50 to 500 μg/mL. Such enhanced uptake can be attributed to the introduction of FA on the surface of the FCPP NPs. As is well known, FA has a high binding affinity for the FA receptor which is frequently overexpressed in human cancer cells. Several studies in the literature confirm that FA modified NPs can improve the chemotherapeutic efficacy of drugs due to their efficient cellular internalization through FA-mediated endocytosis [35]. Together, the results of the cell uptake study indicated that FCPP-NEMs can serve as an efficient carrier for enhanced delivery of a drug to tumor cells, thereby improving the antitumor effects of the encapsulated chemotherapeutic drug.

### 3.6. Cytotoxicity

**Figure 7:**
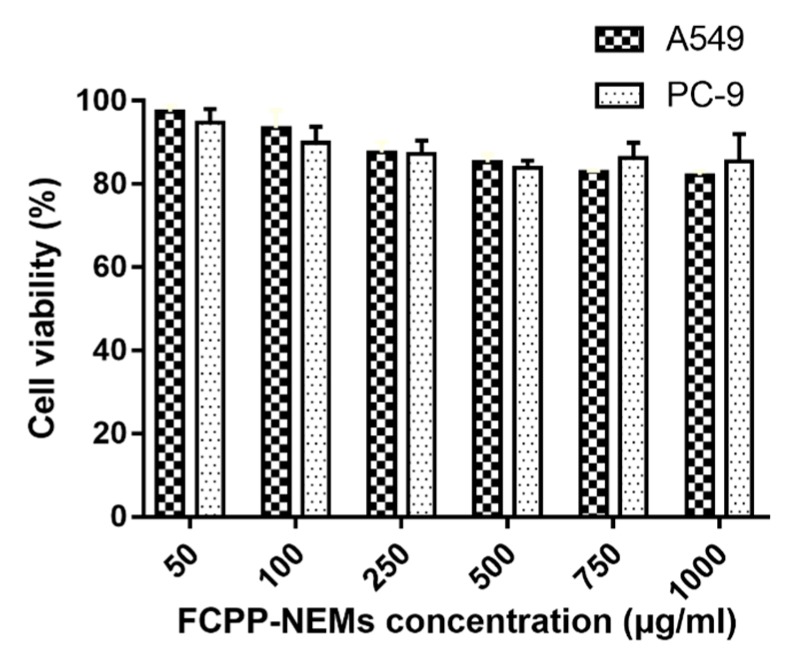
Effect of FCPP-NEMs on A549 and PC-9 cells at various concentrations (*n* = 6).

**Figure 8:**
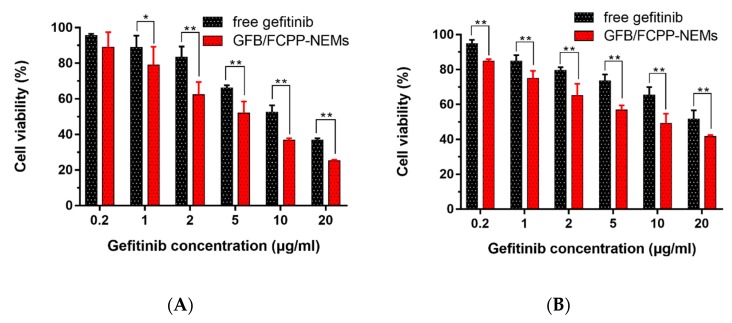
Effect of free gefitinib and GFB/FCPP-NEMs on A549 cells (**A**) and PC-9 cells (**B**) viability at various concentrations (*n* = 6), * *p* < 0.05, ** *p* < 0.01.

After the drug release and cell uptake evaluation, the GFB/FCPP-NEMs may be assumed to display a more enhanced cytotoxic effect than gefitinib crystals. In order to confirm such an assumption, A549 and PC-9 cells were treated with blank FCPP-NEMs, gefitinib crystals, and GFB/FCPP-NEMs at various concentrations for 48 h, and their cytotoxicity was assessed by the MTT colorimetric assay. The results presented in Figure 7 reveal that blank FCPP-NEMs had no apparent inhibitory effect on the proliferation of both A549 and PC-9 cells at the tested concentrations. More than 90% of the cells were viable at concentrations as high as 1000 μg/mL, which indicated that the FCPP-NEMs had little cytotoxic effect on both A549 and PC-9 cells. Since CS, P407, PLGA and β- glucan are FDA-approved biodegradable and biocompatible polymers, FCPP-NEMs could be safely used as a drug delivery system for targeted treatment of lung tumors with chemotherapeutic agents. On the other hand, both free gefitinib and GFB/FCPP-NEMs showed a significant dose- dependent cytotoxicity against A549 cells after incubation for 48 h (Figure 8A). The 50% inhibitory concentrations (IC_50_) for free gefitinib was 8.69 μg/mL, while that for GFB/FCPP-NEMs was decreased by 3.82-fold to 2.27 μg/mL, compared to that of free gefitinib. After encapsulation of gefitinib into FCPP-NEMs, the enhanced cytotoxicity of gefitinib can be mainly attributed to their efficient cellular internalization and pH-dependent prolonged release effect resulting from the encapsulation by FCPP-NEMs. As previously reported, encapsulation of chemotherapeutic agents into NPs or MCs is an effective approach to improve their intracellular delivery, and thereby, enhance their antitumor efficacy [14,16]. For example, Cosco et al. reported that CS-coated PLGA complexes increased the tumor cells uptake of miR-34a, which led to a significant decrease in tumor cell viability [36]. Similarly, GFB/FCPP-NEMs showed an enhanced cytotoxicity to PC-9 cells at the tested concentrations compared with free gefitinib (Figure 8B). The IC_50_ for free gefitinib and GFB/FCPP- NEMs was 10.09 μg/mL and 4.52 μg/mL, respectively. Thus, the prepared FCPP-NEMs system is a promising multifunctional carrier system for improving the antitumor effects of the chemotherapeutic agent gefitinib.

### 3.7. Physicochemical Stability

The particle size, crystallinity, drug content, and related substance of gefitinib were used to evaluate the physicochemical stability of GFB/FCPP-NEMs after storage for 3 months. As shown in Table S3, there were no obvious differences in particle size, drug content, and related substances of gefitinib for the sample of GFB/FCPP-NEMs before and after storage. In addition, the endothermic peak of crystalline gefitinib also cannot be observed in the DSC thermogram of GFB/FCPP-NEMs, suggesting that the prepared GFB/FCPP-NEMs also existed in an amorphous state after storage (Figure S4). Taken together, the GFB/FCPP-NEMs showed an excellent physicochemical stability.

## 4. Conclusions

In this study, a novel FCPP-NEMs-based chemotherapeutic drug delivery system for targeted treatment of lung tumors was designed to afford an FA receptor-mediated tumor targeting property, facilitated intracellular accumulation, pH-triggered sustained release, and enhanced cytotoxicity of the chemotherapeutic agent gefitinib. The prepared GFB/FCPP-NEMs were nearly spherical capsules with a median diameter of around 2.2 μm and a highly porous surface. GFB/FCPP-NEMs can quickly dissociate into their primary particles, GFB/FCPP NPs, after hydration. Besides providing pH- triggered prolonged gefitinib release, the GFB/FCPP-NEMs can facilitate the uptake of the encapsulated cargo by tumor cells. Therefore, the use of GFB/FCPP-NEMs results in a significant improvement in the cytotoxicity of gefitinib compared with the free drug. This study suggests that the developed FCPP-NEMs is a promising carrier system for potential lung tumor-targeted delivery of gefitinib, which is anticipated to improve the antitumor effects of gefitinib and reduce its systemic side effects.

## Figures and Tables

**Table 1:** Characterization of different samples (*n* = 3).

Formulations	Particle Size (nm)	Polydispersity Index	Zeta Potential (mV)	^1^ EE (%)	Drug Loading (%)
P407/PLGA NPs	175.8 ± 11.2	0.08 ± 0.01	−11.74 ± 1.24	——	——
GFB/P407/PLGA NPs	180.0 ± 12.3	0.16 ± 0.05	−12.54 ± 2.30	90.42 ± 0.89	12.96 ± 0.41
GFB/FCPP NPs	255.4 ± 14.5	0.24 ± 0.09	+3.75 ± 1.85	95.57 ± 1.33	7.68 ± 0.36

^1^ EE, entrapment efficiency.

## Supplementary Materials

The following are available online at https://www.mdpi.com/1999-4923/11/9/469/s1, Figure S1: SEM photograph of gefitinib crystals, Figure S2: FT-IR spectra of (**a**) FA-conjugated CS, (**b**) FA, and (**c**) CS, Figure S3: SEM photograph of GFB/FCPP-NEMs after drug release for 2 h, Figure S4: DSC thermogram of GFB/FCPP-NEMs after storage for 3 months, Table S1: Model parameters of the tested samples at pH 5.0, Table S2: Model parameters of the tested samples at pH 7.4, Table S3: Median diameter, span, related substances of gefitinib, and drug content of GFB/FCPP-NEMs before and after storage for 3 months (*n* = 3).
