# Supplementary material for: Development and Evaluation of Multifunctional Poly(Lactic-co-glycolic acid) Nanoparticles Embedded in Carboxymethyl β-Glucan Porous Microcapsules as a Novel Drug Delivery System for Gefitinib
Source: Pharmaceutics. 2019 Sep 12;11(9):469. doi: 10.3390/pharmaceutics11090469 (PMC6781315; doi:10.3390/pharmaceutics11090469)
Supplement: Supplementary file 1 [file pharmaceutics-11-00469-s001.pdf]

pharmaceutics

MDPI

Article

Development and Evaluation of Multifunctional Poly(Lactic-co-glycolic acid) Nanoparticles Embedded in Carboxymethyl β-Glucan Porous Microcapsules as a Novel Drug Delivery System for Gefitinib

Xiaonan Li [1,2], Jinglei Wang [2], Shang Li [1], Zhaorong Liu [2], Zhiru Zheng [2] and Yanzhuo Zhang [1,2,*]

Preparation of FA-conjugated CS

FA, n-hydroxysuccinimide (NHS), and 1-(3-dimethylaminopropyl)-3-ethyl carbonimide hydrochloride (EDC·HCI) (with a molar ratio of 1:1.2:1.2) were dissolved in 10ml of dimethyl sulfoxide, and then stirred for 3 h to activate carboxyl groups of FA. Subsequently, 10 ml of 1% (w/v) CS solution was dipped into the above dimethyl sulfoxide solution, and the mixed solution was stirred at 35°C in the dark for 16 h. Next, the solution was brought to pH 9.0 by addition of diluted aqueous NaOH and dialyzed first against PBS (pH 7.4) for 3 days and subsequently against distilled water for 3 days. Finally, the obtained solution was lyophilized at −55°C for 10 h to collect the FA-conjugated CS.

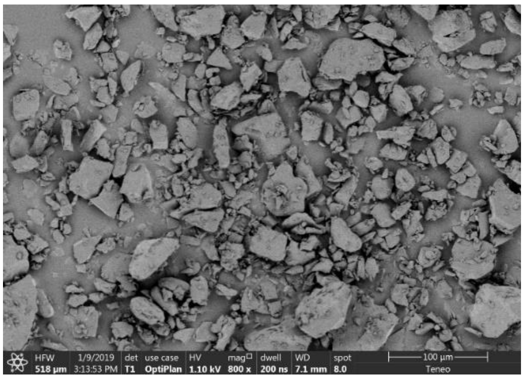

Figure S1. SEM photograph of GFB crystals.

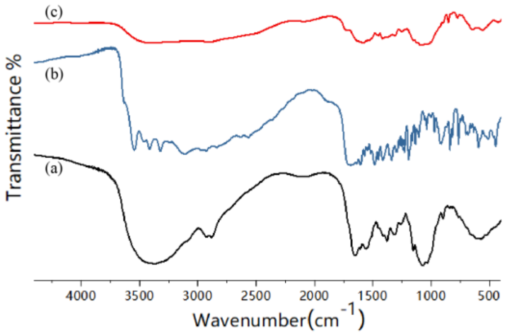

Figure S2. FT-IR spectra of (a) FA-conjugated CS, (b) FA, and (c) CS.

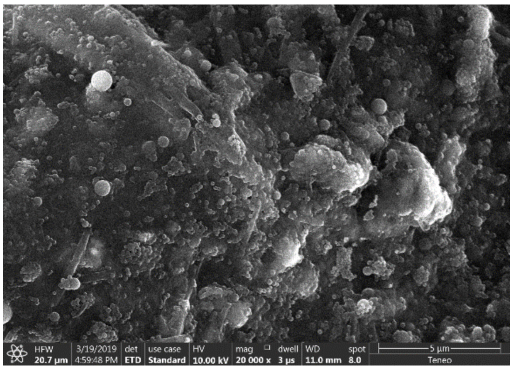

Figure S3. SEM photograph of GFB/FCPP-NEMs after drug release for 2h.

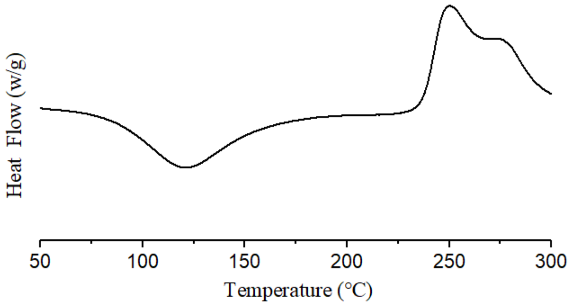

Figure S4. DSC thermograms of GFB/FCPP-NEMs after storage for 3 months.

Table S1. Model parameters of the tested samples at pH 5.0.

Samples	Zero-order		First-order		Higuchi		Korsmeyer-Peppas		
	k	r^2	*k*	r^2	*k*	r^2	*k*	r^2	*n*
GEF/FCPP NPs	0.006	0.107	0.631	0.999	0.066	0.391	0.528	0.961	0.069
GFB/FCPP-NEMs	0.009	0.394	0.232	0.998	0.087	0.705	0.328	0.905	0.207

k is the release rate constant, r^2 is the correlation coefficient, and *n* is the release exponent.

Table S2. Model parameters of the tested samples at pH 7.4.

Samples	Zero-order		First-order		Higuchi		Korsmeyer-Peppas		
	k	r^2	*k*	r^2	*k*	r^2	*k*	r^2	*n*
GEF/FCPP	0.012	0.708	0.082	0.960	0.096	0.876	0.124	0.865	0.439
GFB/FCPP-NEMs	0.009	0.944	0.031	0.971	0.065	0.949	0.031	0.968	0.685

k is the release rate constant, r^2 is the correlation coefficient, and *n* is the release exponent.

Table S3. Median diameter, span, related substances of GFB, and drug content of GFB/FCPP-NEMs before and after storage for 3 months (*n* = 3).

Formulations	Median diameter (µm)	Span	Related substances (%)	Drug content (%)
GFB/FCPP-NEMs[a]	2.2±0.1	1.6±0.2	< 0.6	99.2±0.4
GFB/FCPP-NEMs[b]	2.4±0.1	1.9±0.1	< 0.9	98.6±0.8

GFB/FCPP-NEMs[a] indicates GFB/FCPP-NEMs before storage.

GFB/FCPP-NEMs[b] indicates GFB/FCPP-NEMs after storage for 3 months.
